# Developmental Cascade Effects of Maternal Depression on Offspring Substance Use Across Adolescence: Pathway Through Mother-Child Relationship Quality and Peer Deviancy

**DOI:** 10.1007/s10802-021-00893-y

**Published:** 2022-01-29

**Authors:** Katie J. Paige, Nolan E. Ramer, Craig R. Colder

**Affiliations:** 1 Department of Psychology, University at Buffalo, State University of New York, 359 Park Hall, Buffalo, NY, 14260, USA

**Keywords:** Substance use, Developmental cascade, Maternal depression, Adolescence, Peer deviancy, Mother-child relationship

## Abstract

There is evidence that maternal depression can disrupt adolescent social development and trigger a risk cascade to adolescent substance use that involves poor quality mother-child relationships (Lovejoy et al., 2000) and affiliation with deviant peers (Visser, de Winter, & Reijneveld, 2012). However, relatively little work has considered maternal depression as a catalyst for this risk pathway to adolescent substance use. The current study aims to clarify whether maternal depression has cascading effects to adolescent substance use through related developmental systems. Using structural equation modeling and bootstrapping for testing indirect effects, we tested the prospective association between maternal depression and middle adolescent substance use and whether poor mother-child relationship quality and peer deviancy mediated this relationship. We controlled for a variety of important cofounding variables. The sample included N = 338 adolescents (57% female; predominantly non-Hispanic Caucasian (83.14%) or African American (8.88%)) and mothers drawn from a larger nine-year longitudinal study of adolescent substance use. Data from wave 1 through wave 6 of the longitudinal project were utilized. The average age of adolescents was 11.6, 12.6, 13.6, 14.6, 15.5, and 16.6 at W1-W6, respectively. The indirect effect from maternal depression to substance use was supported (*ab* = 0.03, 95% CI [0.002, 0.07]). Findings emphasize that future work should more closely examine how maternal depression operates in developmental cascade models of adolescent substance use.

## Introduction

Adolescence is a critical period for the development of substance use (SU). Across 8th to 12th grade, past month cannabis use increases from 7% to 22%, past month alcohol use increases from 8% to 29%, and past month cigarette use increases from 2% to 6% (Johnston et al., 2019). These prevalence rates raise concerns due to the short- and long-term adverse consequences of adolescent SU, including a more steeply escalating course of use that is significantly more likely to reach diagnostic thresholds for SU disorders in adulthood (Chassin et al., 2014). For example, adolescents who endorse drinking before 15-years-old have four to six times the rate of lifetime SU disorder compared to individuals who do not initiate alcohol use until age 21 (Grant & Dawson, 1997; Center for Behavioral Health Statistics and Quality, 2015). Moreover, the majority of adults who have a SU disorder started using before age 18, substantiating that adolescent SU represents a significant risk factor for poor psychosocial adjustment in adulthood (Dennis et al., 2002). It is, therefore, critical to identify developmental pathways that confer risk for increases in SU across adolescence. The developmental cascade framework provides a useful approach to study complex and dynamic risk pathways to adolescent SU.

Although many risk factors to adolescent SU have been well-established using this framework, such as externalizing problems, paternal alcoholism, and paternal antisociality, (Eiden et al., 2016; Lynne-Landsman et al., 2010), few studies have examined cascade pathways by which maternal depression may confer risk (Sitnick et al., 2014). This is a notable limitation as maternal depression has been hypothesized to elevate the risk of offspring SU indirectly through ecological determinants, including family factors (Downey & Coyne, 1990), yet such a model has not been widely tested. Moreover, if risk trajectories to adolescent SU involve maternal depression, current prevention efforts may be missing a critical set of intervention targets. The current study aimed to advance the literature by using a developmental cascade framework to examine how maternal depression may contribute to risk trajectories for adolescent SU.

Developmental cascades involve cumulative consequences of interactions and transactions among multiple levels of individual (e.g., psychopathology, gender) and ecological (e.g., family, peers) systems that shape development and adjustment outcomes (Masten & Cicchetti, 2010). Indeed, cascade effects include factors influencing other factors proximally within the same system (e.g., maternal depression impairing mother-child relationship quality) or influence that spreads between systems (e.g., peer deviancy leading to adolescent SU) across time. Examining adolescent SU within a developmental context has been very fruitful.

There is sufficient empirical evidence to suggest that maternal depression may trigger a negative cascade which extends into adolescence, including poor quality mother-child relationships (Lovejoy et al., 2000) and affiliation with deviant peers during a critical period of social development (Ary et al., 1999; Visser et al., 2012). Such a cascade is highly relevant to understanding SU because poor mother-child relationships and peer deviancy precipitate adolescent SU (Ferguson & Meehan, 2011; Visser et al., 2012). Although these individual links are well-established (maternal depression to poor mother-child relationship quality, poor mother-child relationship quality to peer deviancy, and peer deviancy to SU), maternal depression as a catalyst for these family and peer pathways to SU has not yet been tested. This is a significant limitation because clarifying the effects of maternal depression on adolescent SU through related developmental systems will help the field incorporate maternal depression into developmental models of SU and may identify robust intervention targets. We aimed to address this gap.

### Maternal Depression and the Mother-Child Relationship: Risk for Adolescent SU

There is strong empirical evidence that maternal depression is a significant risk factor for a wide range of offspring maladjustment outcomes (Goodman et al., 2011), including child and adolescent externalizing symptoms (Vallotton et al., 2016). Yet, results from past work have not consistently supported direct associations between maternal depression and offspring SU (Cortes et al., 2009; Nomura et al., 2002). Moreover, the few longitudinal studies that have investigated the relationship between maternal depression and adolescent SU have failed to control for prior levels of SU (Sitnick et al., 2014), or have not considered family and peer factors as potential mediators (e.g., Cortes et al., 2009). These are significant omissions, as considering relevant mediational pathways may help clarify this mixed literature and identify specific risk pathways to adolescent SU.

Downey and Coyney (1990) proposed that maternal depression may indirectly elevate the risk of offspring externalizing problems, including SU, through family dysfunction. Indeed, maternal depression leads to poor mother-child relationship quality (Gerdes et al., 2007), negative parenting behavior (Hendricks & Liu, 2012), and disengagement from the child (Lovejoy et al., 2000). These maladaptive parenting behaviors are thought to underlie associations between maternal depression and offspring externalizing symptoms (Foster et al., 2008; Hendricks & Liu, 2012). Importantly, poor mother-child relationship quality, characterized by low warmth, low support, and distance, prospectively predicts escalations in adolescent drinking (Visser et al., 2012). In fact, a review suggested that among family-related risk factors for adolescent SU, poor mother-child relationship quality has received especially strong support (Hummel et al., 2013).

These findings underscore the importance of examining the role of maternal depression in developmental cascade models of adolescent SU, and suggest that maternal depression may operate on adolescent SU through a cascade involving poor mother-child relationship quality. “The quality of all dyadic relationships is influenced by individual characteristics of the relationship partners plus their corresponding interaction history” (Asendorpf, 2002). It is therefore not surprising that mother-child relationship quality is an interpersonal dynamic that is stable across long periods of time (Bornstein & Putnick, 2021) and influenced proximally by changes in the individual characteristics. Mother-child relationship quality is potentially compromised when mothers are dealing with psychological distress, ranging from subclinical to impaired levels (Oyserman et al., 2000). Fluctuations in depression symptoms are common, and when a mother experiences increases in depression symptoms, she may withdraw, be less sensitive to the feelings and moods of her child, and display lower levels of warmth (Oyserman et al., 2000). That is, maternal depression likely operates on mother-child relationship quality proximally, and empirical evidence supports this notion, as maternal depression and poor mother-child relationship quality typically co-occur within-time (Lovejoy et al., 2000). Although poor mother-child relationship quality is important, it alone may not be sufficient to understand how maternal depression increases risk for adolescent SU. Indeed, adolescent SU typically occurs in the peer context (Ennett et al., 2006), and peer deviancy is one of the most robust precipitants of adolescent SU (Chassin et al., 2010). Therefore, examination of pathways to adolescent SU must also consider the peer system.

### The Transition from Parents to Peers

Adolescence is a period of elevated responsiveness to social reward, and engenders a heightened desire to affiliate with peers (Spear, 2000). Additionally, peers are thought to become increasingly influential during adolescence (Harris, 1995; Ferguson & Meehan, 2011) and prior work suggests that adolescents are highly susceptible to deviant peer influence on SU (Ferguson & Meehan, 2011; Fergusson, et al., 2002). Deviant peers provide support and reinforcement for SU (Albert et al., 2013; Logue, et al., 2014), and access to drugs and alcohol (Gilligan et al., 2012).

Given the deviant peer context is a proximal influence on adolescent SU, understanding developmental pathways to affiliation with deviant peers is important. During this critical period of social development there are likely spillover effects, whereby quality of mother-child relationships precipitates affiliation with deviant peers (Ingoldsby et al., 2006; Van Doorn et al., 2011). Indeed, theory and research suggest that interpersonal experiences with parents serve as working models for the formation and maintenance of adolescent friendships (De Goede et al., 2009; Furman et al., 2002). Taken together, adolescents who have poor relationships with their parents, characterized by distance and conflict, may model learned negative interpersonal skills in their peer relationships, leading to rejection from prosocial peers and affiliation with deviant adolescents (Chen et al., 2015; Dishion et al., 1991). Accordingly, parent and peer influences are interconnected in risk pathways to adolescent SU (Nickerson & Nagle, 2005).

### Covariates

Informed by extant research, the current study aimed to address a gap in the literature by testing a risk pathway from maternal depression to adolescent SU involving poor mother-child relationship quality and peer deviancy. There are myriad other individual and contextual factors that may serve as confounding variables when testing the proposed cascade and it is not possible to include all possible pathways from potential confounders to our theoretical variables of interest. Accordingly, we considered the literature to decide on potential confounding variables and their role in our proposed cascade model. Maternal SU and antisocial behavior, and adolescent depression and externalizing symptoms may disrupt a healthy mother-child relationship (Crowell et al., 1991; Leonard & Eiden, 2007). Therefore, these variables may confound our hypothesized association between maternal depression and poor mother-child relationship quality, so we included these variables as statistical controls when predicting mother-child relationship quality. Additionally, adolescent externalizing symptoms and maternal antisocial behaviors may confound associations between poor mother-child relationship quality and peer deviancy (Brook et al., 2011; Frick et al., 1992). Therefore, we included these variables as statistical controls when predicting peer deviancy. As maternal depression is also associated with externalizing problems, we controlled for its association with maternal SU and antisocial behaviors. Adolescent gender was also included as a statistical control variable given prior work suggesting associations gender and both mother-child relationship quality and peer deviancy (Brook et al., 2011; Mandara et al., 2012). Finally, important demographic variables were included as statistical control variables (e.g., minority status and maternal education).

### The Current Study

Relatively little extant research has examined intermediary processes that may account for the link between maternal depression and offspring SU (Cortes et al., 2009; Sitnick et al., 2014), which is a significant limitation given that maternal depression has been hypothesized to elevate the risk of offspring SU indirectly through ecological determinants (Downey & Coyne, 1990). Moreover, developmental models of SU have focused on paternal antisociality and alcoholism, which may leave a critical set of intervention targets involving maternal depression understudied. Both the quality of the mother-child relationship and peer deviancy are well-established prospective correlates of adolescent SU (Ferguson & Meehan, 2011; Hummel et al., 2013), and we proposed that it is important to consider both in order to understand risk of maternal depression for adolescent SU. Finally, developmental cascade models of SU often fail to account for stability of SU across time (Sitnick et al., 2014) and theoretically important confounding variables. The current study tested a risk pathway from maternal depression to adolescent SU involving poor mother-child relationship quality and affiliation with deviant peers, statistically controlling for potential confounding variables. Our longitudinal design and assessment of multiple systems of development provided a rigorous test of a developmental risk pathway to adolescent SU (Masten & Cicchetti, 2010).

### Hypotheses

**Fig. 1:**
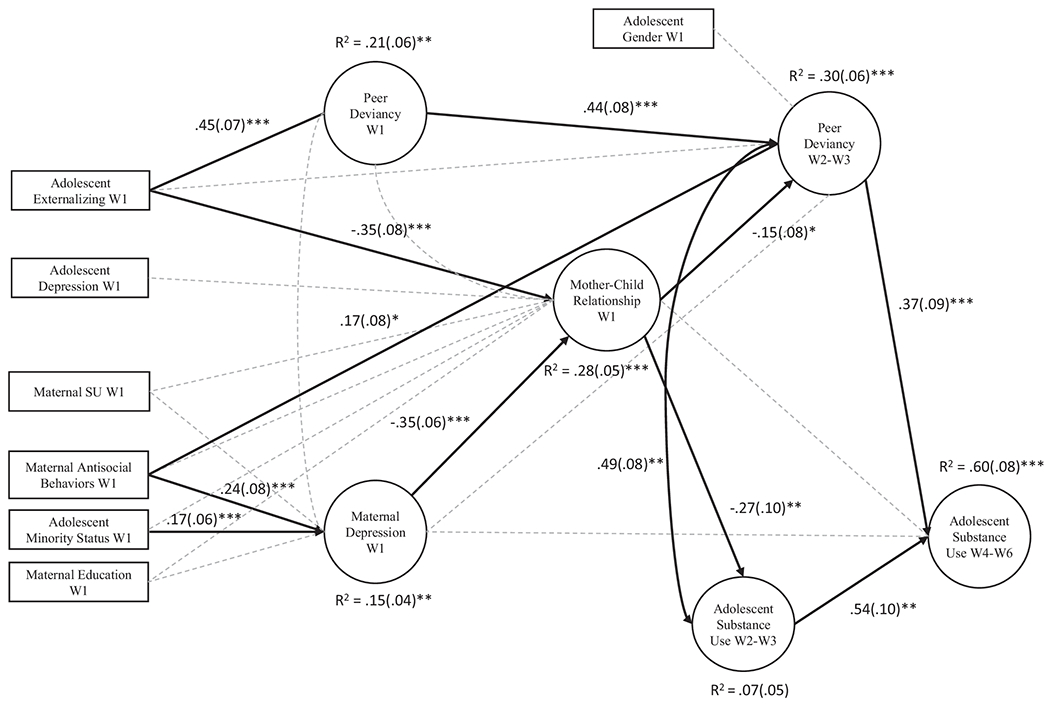
Structural Model of Cascade Effect from Maternal Depression to Adolescent Substance Use. Solid black lines are significant and dotted grey lines are non-significant pathways. Betas are reported next to significant associations and standard errors are reported in parentheses. Levels of significance were based on unstandardized regression estimates. *W* Wave. *p* < 0.05 = *, *p* < 0.01 = **, *p* < 0.001 = ***

We hypothesized that at adolescent age 11, high levels of maternal depression would be concurrently related to poor mother-child relationship quality, which would be prospectively associated with high levels of peer deviancy at ages 12-13, in turn predicting high levels of SU at ages 14-16 (Fig. 1).

## Method

### Participants

The sample of 387 adolescents and their mothers was drawn from a longitudinal study of adolescent SU and recruited using random-digit dialing procedures. Adolescents were eligible if they were between the ages of 11 and 12 at recruitment and did not have any disabilities that would preclude them from either understanding or completing the assessment. The recruitment period ranged from April 2007 to February 2009.

Data from Wave (W) 1 through W6 of the longitudinal project were utilized (of 9 annual waves total). A small number (n=49) of our caregivers were fathers and they were excluded because we did not have sufficient group sizes to compare the effects of maternal and paternal depression symptoms. Therefore, our final sample size for the current study was *N*=338. The average age of participants was 11.6, 12.6, 13.6, 14.6, 15.5, and 16.6 at W1-W6, respectively. The adolescent sample was 57% female and predominantly non-Hispanic Caucasian (83.14%) or African American (8.88%). Median family income at W1 was $70,000 and 6% of families received public assistance income (*for details about the sample, see* Trucco et al., 2014). Overall retention across assessments was strong, ranging from *N*=321 (95%) to *N*=331 (98%).

### Procedure

Interviews at W1-W3 were conducted annually in university research offices. Consent from caregivers and assent from adolescents were obtained prior to participation. Caregivers and adolescents were interviewed in separate rooms to ensure privacy. At W1-W3, families were compensated $75, $85, and $125, respectively.

Annual assessments at W4-W6 involved a brief telephone administered audio-Computer Assisted Self Interview of adolescent SU. Parents provided consent over the phone and were given a phone number and PIN for the adolescent. Adolescent assent was obtained at the initiation of the survey. The interview took approximately 10-15 minutes to complete and families were compensated with $15 gift cards at W4 and W5, and $20 gift cards at W6.

The study was approved by the University at Buffalo institutional review board.

### Measures

All data and codebooks with items used in the study are publicly available at Inter-university Consortium for Political and Social Research.

#### Adolescents

##### SU (W2-W6).

SU was measured with questions assessing (1) past year frequency of alcohol, cannabis, and cigarette use, and (2) past year quantity of alcohol and cigarette use, taken from the National Youth Survey (NYS; Elliott & Huizinga, 1983). Cannabis quantity was not assessed. As such, a quantity by frequency (QxF) index was created for past year alcohol and cigarette use while past year frequency of cannabis use was used as an indicator of cannabis use at each wave.

##### Peer Deviancy (W1-W3).

Peer deviancy was assessed with 15 items from Fergusson and colleagues (1999). Adolescents reported whether any of their three closest friends engaged in a variety of deviant behaviors using a “yes” or “no” response option. Items were bundled into parcels and then summed at each wave, creating four indicators for each latent factor of peer deviancy. Reliabilities for W1 through W3 ranged from good to excellent (Cronbach’s α = 0.79-0.87).

##### Externalizing Symptoms (W1).

Externalizing symptoms were included as a statistical control and assessed using items from the youth self-report form (YSR) of the Achenbach System of Empirically Based Assessment (ASEBA; Achenbach & Rescorla, 2001). Rule-breaking and aggressive behavior items were averaged to form a scale score (Cronbach’s α = 0.85). SU items were removed to avoid confounding with SU variables measured by the NYS.

##### Adolescent Depression (W1).

Adolescent depression symptoms were included as a statistical control variable and assessed with items from YSR of the ASEBA (Achenbach & Rescorla, 2001). A scale has been derived on the YSR that corresponds to the DSM category for depression which demonstrates better sensitivity, predictive power, and discriminant validity than the original internalizing scales (Lengua et al., 2001). Items were averaged to create a scaled score of depression symptoms (Cronbach’s α = 0.68).

#### Caregiver

##### Maternal Depression (W1).

Maternal depression was assessed using the Center for Epidemiological Studies Depression Scale (CES-D; Radloff, 1977). All 20 items from the CES-D were randomly placed into four parcels, which were then averaged to create indicators for the maternal depression latent factor (Cronbach’s α = 0.91).

##### Mother-child Relationship Quality (W1).

Mother-child relationship quality was assessed using the Parent-Child Relationship scale of the Loeber Youth Questionnaire (LYQ; Jacob et al., 2000). Informed by empirical evidence suggesting that maternal depression likely impacts mother-child relationship quality in proximal interactions (Lovejoy et al., 2000), we focused on W1 mother-child relationship quality to test concurrent associations with maternal depression. A latent variable for mother-child relationship quality at W1 was specified using 16 items from the scale to create four randomly parceled indicators (Cronbach’s α = 0.82).

##### Maternal Antisocial Behavior (W1).

Lifetime maternal antisocial behavior was included as a statistical control variable and assessed using the Antisocial Behavior Checklist (Wong et al., 1999). Items were averaged to create a scaled score (Cronbach’s α = 0.74).

##### Maternal SU (W1).

Similar to adolescent alcohol use, a QxF index of number of drinks in the past year was calculated using quantity and frequency items from Cahalan and colleagues (1969). We utilized a dichotomous variable to assess current cigarette use (1=No, 2=Yes). Maternal cannabis use was not assessed. To reduce model parameters, items were standardized and a mean score for maternal alcohol and cigarette use was calculated.

##### Demographic Covariates (W1).

Caregivers reported on their child’s gender, which was coded 0 for male and 1 for female, and minority status^1^ (0=Caucasian/non-Hispanic white, 1=Minority). Mothers also reported on their own education level, which was coded from 1=Grade school to 7=Graduate/Professional school.

### Data Analytic Strategy

Structural equation modeling (SEM) with Robust Maximum Likelihood estimation (MLR) was used to test the proposed prospective pathways using Mplus 8.2 (Muthén & Muthén, 1998-2018). Monte Carlo simulations were used to determine power for the mediational pathway (Muthén & Muthén, 2002). Using our sample size of *N*=338, results revealed we had adequate power (> 0.80) to detect small effect sizes across each link in our mediational chain. When there is power to detect each link in a mediational chain, there is adequate power to detect the mediated effect (Cohen, 1988). Given non-normality of the observed SU variables (Table 1), these variables were log-transformed (Tabachnick & Fidell, 2013). The log-transformed variables were still skewed (alcohol skew = 0.94-5.46; cannabis skew = 2.20-8.21; cigarette skew = 3.24-6.73) and had high kurtosis (alcohol kurtosis = −0.45-31.86; cannabis kurtosis = 3.96-69.03; cigarette kurtosis = 10.21-45.42) and thus MLR was used to accommodate for the remaining non-normality in observed variables. Significance of indirect effects was tested using bootstrapping with 10,000 samples and 95% confidence intervals (Shrout & Bolger, 2002). Indirect effect sizes were estimated using Lachowicz and colleagues (2018) υ, which is estimated by squaring the standardized indirect effect and is interpreted as the proportion of variance explained by the indirect effect.

Model building occurred in two steps: measurement models were estimated and then combined into structural models (Kline, 2015). First, single factor measurement models for each latent variable were estimated (*see* Supplemental Materials 1 for information on measurement models’ fit). To reduce the number of model parameters, items were parceled as described above (Little et al., 2013), and our data were collapsed across waves with consideration of developmental periods during adolescence and a focus on testing the proposed mediational chain. As expected, rates of use were very low at W1 for alcohol (2.06% endorsed past year drinking), cigarette (0.59% endorsed past year cigarette use), and cannabis (0% endorsed past year cannabis use) given the age of the sample. Accordingly, we constructed the latent variables for SU beginning at W2. We collapsed SU at W2 and W3 to represent a latent variable for early adolescent SU, as rates of SU increase dramatically after W3. SU was also collapsed across W4-W6 in order to create a latent variable that is representative of middle adolescent SU, our outcome of interest. W2-W3 and W4-W6 single factor SU measurement models were constructed with each substance (alcohol, cannabis, and cigarette) serving as an indicator. The early adolescent variable for peer deviancy was constructed in a similar fashion (e.g., at W2-3) in order to control for confounding associations with early adolescent SU. Given that there was endorsement of peer deviancy at W1, which would be expected during this developmental period, it was important to control for the stability of peer deviancy when testing the hypothesized association between mother-child relationship quality at W1 and peer deviancy at W2-3. Therefore, we also created a latent variable for peer deviancy at W1. Next, a structural path model was constructed (Fig. 1). The hypothesized prospective pathways and within-time covariances were first specified and evaluated for model fit. Next, covariates were added. Modification indices were used to add paths that provided significant improvement in fit and were consistent with theory (Schreiber, 2017).

Model fit was assessed using conventional absolute and incremental SEM fit indices. Since cutoffs for “good” fit can vary between models, ranges were used to determine acceptability of model fit (Hu & Bentler, 1999; Marsh et al., 2004). These fit indices and ranges included chi-square estimates of fit (a significant chi-square indicates poor fit), the comparative fit index (CFI) and Tucker-Lewis index (TLI; for both < 0.90 is poor, 0.90 to 0.94 is acceptable, and 0.95 or greater is excellent), root mean square error approximation (RMSEA; > 0.08 is poor, 0.05 to 0.07 is acceptable, and 0.05 or lower is excellent), and standardized root mean square residual (SRMR; SRMR, > 0.09 is poor, 0.06 to 0.09 is acceptable, and 0.06 or lower is excellent). Nested chi-square difference tests and changes in CFI (changes of 0.01 or greater; Cheung & Rensvold, 2002) were used to assess support for model modifications.

## Results

### Descriptives

Descriptive statistics and bivariate correlations for observed variables are reported in Tables 1 and 2. Bivariate correlations between latent factors are reported in Supplemental Materials 1. At W2, adolescents reported drinking less than one drink in the past year on average (i.e., sipping). By W6, adolescents on average reported drinking approximately 20 drinks in the past year. Adolescents’ average past year cannabis use increased from less than 1 time at W2 to about 7 times at W6. Average past year cigarette use increased from less than one cigarette at W2 to approximately 30 cigarettes at W6. These descriptive statistics suggest that we are examining the early stages of adolescent SU, as would be expected given the age of the sample.

### Measurement Models

Single factor models all provided adequate fit to the data (Supplemental Materials 1). The combined measurement model provided good fit (χ^2^ (df) = 235.77 (193), p = 0.02, CFI = 0.98, TLI = 0.98, RMSEA = 0.03, SRMR = 0.05).

### Structural Equation Models

Next, prospective paths and within-time covariances replaced factor covariances in the combined measurement model to test study hypotheses. For more details on model specification, see Supplemental Materials 1.

#### Structural Model

The final structural model provided good fit (χ^2^ (df) = 453.94 (334), *p* < 0.001, CFI = 0.96, TLI = 0.95, RMSEA = 0.03, SRMR = 0.06). Results for the path model are reported in Fig. 1. Regarding control variables, maternal antisocial behavior was positively and cross-sectionally related to maternal depression, such that mothers who endorsed more lifetime antisocial behavior experienced higher levels of depression^2^. At W1, high levels of externalizing symptoms were associated with poorer mother-child relationship quality. Adolescents who exhibited high W1 externalizing symptoms were more likely to affiliate with deviant peers at W1, but externalizing symptoms were unrelated to peer deviancy prospectively. Additionally, high maternal antisocial behavior was prospectively related to elevated W2-W3 peer deviancy. Finally, minority status was associated with high levels of maternal depression.

Regarding study hypotheses, elevated maternal depression in early adolescence was significantly associated with low levels of mother-child relationship quality, and poor mother-child relationship quality prospectively predicted greater affiliation with deviant peers in early adolescence (W2-W3). W2-W3 peer deviancy, in turn, robustly predicted elevated levels of W4-W6 SU.

### Cascade Effects from Maternal Depression to Adolescent SU: Mediation

The hypothesized indirect effect from maternal depression to middle adolescent SU was supported (*ab* = 0.03, 95% CI [0.002, 0.07]). Elevated maternal depression was associated with poor mother-child relationship quality, which was prospectively associated with increased peer deviancy, which in turn, predicted increased SU. This indirect effect was small and explained 0.1% of the variance in middle adolescent SU (υ = 0.001). The indirect effect from maternal depression to middle adolescent SU through poor mother-child relationship quality and high early adolescent SU was also supported (*ab* = 0.05, 95% CI [0.026, 0.196]). This indirect effect was small and explained 0.3% of the variance in middle adolescent SU (υ = 0.0025). Total variance explained in W4-W6 adolescent SU was high (R^2^ = 0.60), suggesting the model accounted for a substantial portion of variability in middle adolescent SU.

## Discussion

There has been limited work investigating the ways in which maternal depression may confer risk for adolescent SU (Sitnick et al., 2014), and previous studies which have examined direct associations have yielded mixed results (Cortes et al., 2009; Nomura et al., 2002). Notably, maternal depression may trigger a cascade of negative sequela for adolescent development, including poor mother-child relationship quality (Lovejoy et al., 2000) and affiliation with deviant peers (Ary et al., 1999), both of which are robust predictors of adolescent SU (Ferguson & Meehan, 2011; Shelton & van den Bree, 2010). We propose that some of the confusion in this literature is due to a failure to examine links between maternal depression and adolescent SU using a developmental cascade framework and considering potential mediators. In this study, we aimed to address these limitations by using a longitudinal design and assessment of multiple systems of development which contribute to adolescent SU. Specifically, we tested the cascading effects from maternal depression to adolescent SU through poor mother-child relationship quality and peer deviancy. Importantly, our study was able to control for prior levels of SU and include several theoretically important statistical control variables (maternal antisocial behavior and SU, and adolescent externalizing and depression symptoms) that may confound hypothesized associations.

### Developmental Cascade Effects: Mediation

There was support for our hypothesis that mother-child relationship quality and peer deviancy would mediate the relationship between maternal depression and adolescent SU. This substantiates that developmental cascade models may be a fruitful means by which to understand the role that maternal depression plays in the etiology of adolescent SU (Maten & Cicchetti, 2010; Sitnick et al., 2014). While not a hypothesized mediational pathway, the indirect effect from maternal depression to middle adolescent SU through poor mother-child relationship quality and high early adolescent SU was also supported. These findings highlight that maternal depression and poor mother-child relationship quality carry significant risk for SU in both early and middle adolescence. Indeed, the significant relationships we found across multiple systems of development highlight several possible points of intervention to prevent adolescent SU which are discussed in more detail below.

### Effects of Maternal Depression on Mother-Child Relationship Quality

In early adolescence (ages 11-12), high levels of maternal depression were related to low levels of mother-child relationship quality. This corroborates findings from previous studies that maternal depression is associated with negative parenting behavior and disengagement from offspring (Lovejoy et al., 2000). Associations between maternal depression and negative parenting behaviors are thought to reflect the affective, cognitive, and physical symptoms that characterize depression; mothers who are irritable, have poor concentration, and are fatigued may express more negative affect toward their children, be less attentive, and be less tolerant of normative child behavior (Lovejoy et al., 2000). Notably, research on the effects of maternal depression on aspects of mother-child relationship quality has been concentrated in infancy and childhood (e.g., Lovejoy et al., 2000; Martins & Gaffan, 2000), with a smaller number of studies using adolescent samples (Kim et al., 2015). Our study further supports the co-occurrence of maternal depression and poor relationship quality with offspring during early adolescence.

### Prospective Effects of Mother-Child Relationship Quality on Peer Deviancy

Low levels of mother-child relationship quality prospectively predicted affiliation with deviant peers in early adolescence (ages 12-13). While there is less longitudinal work examining this relationship, findings from prior cross-sectional studies suggest that children and early adolescents who view relationships with parents as less secure are more likely to affiliate with deviant peers to fulfill attachment needs (Nickerson & Nagle, 2005). Our results expand upon these findings and support theories of spillover effects, which posit that interpersonal experiences with parents serve as working models for the formation and maintenance of adolescent friendships (De Goede et al., 2009; Furman et al., 2002). Therefore, adolescents may learn negative interpersonal skills from poor relationships with parents, and use these maladaptive skills in their peer relationships, leading to rejection from prosocial peers and affiliation with deviant adolescents (Chen et al., 2015; Dishion et al., 1991).

### Prospective Effects of Peer Deviancy on SU

High levels of peer deviancy at ages 12-13 robustly predicted high levels of SU at ages 14-16. This corroborates findings from previous studies that have shown strong correlations between affiliation with deviant peers and SU (Allen et al., 2003; Fergusson et al., 2002).

### Limitations

Conclusions from the current study should be interpreted in the context of certain limitations. Our study excluded fathers. On the one hand, this is sensible as women more commonly experience depression symptoms (Piccinelli & Wilkinson, 2000). However, SU and antisocial behavior are higher among men (Trull et al., 2010; World Health Organization, 2018), and therefore, our developmental cascade from caregiver depression to adolescent SU may operate differently in fathers. Due to the large number of parameters in our complex latent model and the limited number of fathers in the study, we elected to exclude fathers and we could not examine multiple group comparisons by gender. It will be important for future work to consider paternal depression.

Although we used a longitudinal design, the link between maternal depression and mother-child relationship quality was cross-sectional. Maternal depression is by nature episodic (Oyserman et al., 2000), and influences interpersonal dynamics between mothers and children proximally (Lovejoy et al., 2000). Indeed, our measure of maternal depression assessed past month symptoms reflecting short-term, episodic fluctuations. When a mother experiences increases in depression symptoms, she may withdraw, be less sensitive to the feelings and moods of her child, and display lower levels of warmth. Therefore, we expected the impact of fluctuations in maternal depression to compromise the mother-child relationship more proximally than could be captured in our annual assessments. Accordingly, we elected to examine the within-time association between maternal depression and mother-child relationship quality. Still, this choice limited our ability to establish temporal precedence and also to consider possible reciprocal relations between maternal depression and quality of mother-child relationship. A useful direction for future research would be to utilize prospective short-term designs that might better capture proximal dynamics between maternal depression and mother-child relationship quality (e.g., Ecological Momentary Assessment studies).

Relatedly, in some cases we were able to examine associations across reporters (e.g., mother-child relationship quality was assessed using parent-report, while peer deviancy was assessed with adolescent-report), which reduces concerns about inflation of associations due to shared reporter. However, this was not true for all tested associations (e.g., peer deviancy and adolescent SU were both adolescent reported). Future studies may aim to utilize a more fully multimethod assessment approach (e.g., biological verification of SU and observation measures of parent-child relationship quality) along with questionnaire assessments to reduce inflation of associations due to mono-method and single reporter assessment.

Finally, it should be noted that the hypothesized indirect effect constituted a small effect size (2% = small; Cohen, 1988). However, the effect size in the current study was calculated for indirect effects across a five-year-long period of time. Therefore, traditional cutoffs, which were established for cross-sectional studies that do not account for stabilities or time-lagged effects, may result in an underestimation of cross-lagged and indirect effect sizes (Adachi & Willoughby, 2015; Lachowicz et al., 2018). Indeed, small effects, even those below the traditional cutoffs for small effects, may still be meaningful when found above and beyond stabilities, within-time covariances, and covariates, especially when consistent with theoretical expectations (Adachi & Willoughby, 2015). Future work should look to establish empirically supported effect size cutoffs for longitudinal and indirect effect sizes.

### Conclusion and Implications

Findings from the current study emphasize that maternal depression may trigger a cascade to offspring adolescent SU involving poor mother-child relationships and peer deviancy, controlling for several theoretically-important covariates. This is critical, as adolescent SU elevates risk for later SU disorders (Chassin et al., 2014), and better understanding developmental pathways to early SU may aid in prevention efforts. Evidence for a risk trajectory from maternal depression to adolescent SU supports the importance and effectiveness of treating depression in parents to improve child adjustment and SU in particular (Gunlicks & Weissman, 2008). Furthermore, positive associations between peer deviancy and adolescent SU corroborate calls to design better interventions that aim to promote affiliations with prosocial peers (Gifford-Smith et al., 2005). Our study also suggests that affiliation with deviant peers may be attenuated through promotion of positive mother-child relationships, and future intervention efforts should explore this novel possibility.

Finally, our results emphasize the utility of adopting a developmental cascade perspective for research in this literature. Developmental cascade models of adolescent SU go above and beyond identifying unique effects; they represent a useful framework in which to organize risk and protective trajectories involving multiple systems across time. Our study suggests that maternal depression is involved in a risk cascade to adolescent SU.

## Supplementary Material

1776786_Sup_material

## Figures and Tables

**Table 1:** Descriptive Statistics of Study Variables

Variables	Mean	SD	Skew	Kurtosis
W2 Alcohol Use QxF	0.50	3.17	7.99	66.37
W3 Alcohol Use QxF	1.73	8.60	6.25	38.66
W4 Alcohol Use QxF	4.20	12.48	4.05	17.16
W5 Alcohol Use QxF	11.50	36.05	4.07	16.24
W6 Alcohol Use QxF	21.36	52.17	3.33	10.78
W2 Cannabis Use Frequency	0.09	0.81	9.74	100.72
W3 Cannabis Use Frequency	0.60	2.60	5.36	30.07
W4 Cannabis Use Frequency	1.77	7.99	5.48	30.58
W5 Cannabis Use Frequency	4.48	19.80	5.20	26.32
W6 Cannabis Use Frequency	6.67	23.74	4.44	19.38
W2 Cigarette Use QxF	0.04	0.29	7.16	51.36
W3 Cigarette Use QxF	3.06	19.79	7.70	60.81
W4 Cigarette Use QxF	3.00	23.77	12.09	164.63
W5 Cigarette Use QxF	13.37	90.68	9.69	109.40
W6 Cigarette Use QxF	29.56	196.00	9.88	112.86
Maternal Depression	0.80	0.48	0.79	0.16
Mother-child Relationship Quality	2.76	0.22	−1.43	2.64
W1 Peer Deviancy	0.03	0.29	0.95	9.02
W2 Peer Deviancy	0.03	0.39	0.33	3.69
W3 Peer Deviancy	0.03	0.42	0.21	2.78
Adolescent Externalizing	0.21	0.16	1.24	2.10
Adolescent Depression	0.21	0.21	1.32	2.44
Maternal Antisociality	18.51	3.09	2.22	7.03
Maternal Alcohol Use QxF	14.17	6.69	1.47	3.97
Maternal Cigarette Use	1.23	0.42	1.32	−0.25
Adolescent Gender	0.58	0.49	−0.32	−1.91
Adolescent Minority Status	0.17	0.37	1.77	1.13
Maternal Education	5.40	1.35	−0.85	−0.17

*W* Wave, *QxF* Quantity by Frequency

**Table 2:** Zero Order Correlations of Latent Variables and Observed Statistical Control Variables

Variables	1	2	3	4	5	6	7	8	9	10	11	12	13
1. Maternal Depression	-												
2. Mother-child Relationship	−0.39	-											
3. W1 Peer Deviancy	0.01	−0.18	-										
4. W2-W3 Peer Deviancy	0.14	−0.27	0.46	-									
5. W2-W3 Substance Use	0.11	−0.27	0.05	0.47	-								
6. W4-W6 Substance Use	0.02	−0.20	0.20	0.61	0.70	-							
7. Adolescent Gender	−0.001	0.05	−0.08	−0.04	−0.02	−0.02	-						
8. Maternal Antisociality	0.32	−0.25	0.09	0.27	0.07	0.11	0.02	-					
9. Adolescent Depression	0.06	−0.18	0.24	0.14	0.05	0.07	−0.09	0.18	-				
10.Adolescent Externalizing	0.07	−0.37	0.45	0.24	0.10	0.14	−0.18	0.20	0.53	-			
11. Maternal Substance Use	0.20	−0.12	0.07	0.13	0.03	0.05	−0.09	0.34	0.03	0.15	-		
12. Adolescent Minority Status	0.24	0.08	0.02	0.12	0.02	0.04	0.03	0.22	0.03	0.04	0.15	-	
13. Maternal Education	−0.20	0.16	−0.05	−0.19	−0.04	−0.08	0.02	−0.31	−0.09	−0.10	−0.34	−0.20	-

Correlations greater than or equal to |0.10| in magnitude are significant at p < 0.05

*W* Wave

## Data Availability

The data and codebooks for this project are publicly available at Inter-university Consortium for Political and Social Research.

## Footnotes

1 We also tested a model using maternal minority status as a covariate instead of adolescent minority status. Consistent with results from the primary model using adolescent minority status, maternal minority status was not significantly associated with our proposed mediators (mother-child relationship quality and W2-3 peer deviancy), and the hypothesized indirect effect from maternal depression to middle adolescent SU through mother-child relationship quality and peer deviancy was still supported (ab = 0.03, 95% CI [0.002, 0.07]).

2 To examine the possibility that maternal SU was confounding hypothesized associations, we tested a model in which adolescent SU at W2-3 and W4-6 was regressed on maternal SU at W1. The associations were nonsignificant, and the hypothesized indirect effect from maternal depression to middle adolescent SU through mother-child relationship quality and peer deviancy was still supported (*ab* = 0.03, 95% CI [0.002, 0.07]).
