# Supplementary material for: Developmental Cascade Effects of Maternal Depression on Offspring Substance Use Across Adolescence: Pathway Through Mother-Child Relationship Quality and Peer Deviancy
Source: Res Child Adolesc Psychopathol. Author manuscript; Available in PMC 2023 Jul 1. (PMC9246831; doi:10.1007/s10802-021-00893-y)
Supplement: 1776786_Sup_material [file NIHMS1776786-supplement-1776786_Sup_material.docx]

Journal Name: Research on Child and Adolescent Psychopathology

Developmental Cascade Effects of Maternal Depression on Offspring Substance Use Across Adolescence: Pathway Through Mother-Child Relationship Quality and Peer Deviancy

**Supplemental Materials 1**

**Single Factor Measurement Models**

Final fit of single factor measurement models for W1 maternal depression (χ^2^ (df) = 2.88 (2), *p* = .24, CFI = 1.00, TLI =1.00, RMSEA =.04, SRMR =.01), W1 mother-child relationship quality (χ^2^ (df) = 2.69 (2), *p* = .26, CFI = 1.00, TLI = 1.00, RMSEA =.00, SRMR =.01), W1 peer deviancy (χ^2^ (df) = 0.49 (2), *p* = .78, CFI = 1.00, TLI = 1.00, RMSEA =.01, SRMR =.02) and W2-W3 peer deviancy (χ^2^ (df) = 0.31 (1), *p* = .58, CFI = 1.00, TLI =1.00, RMSEA =.00, SRMR =.002) provided excellent fit of the data. Modification indices suggested allowing errors between W2 peer deviancy to covary, which resulted in significant improvement of model fit (*p* < .001). The single factor measurement models for W2-W3 and W4-W6 substance use were just identified. Unit loading identification (ULI) was used to scale latent variables to the metric of the first indicator (Kline, 2015).

**Correlated Measurement Model**

The single factor measurement models were combined into a single correlated measurement model, where all factors were allowed to covary. Factor structure and loading magnitudes are displayed in Figure 1 (*see* Model 1). Inter-factor correlations are reported in Table 1. No modifications were made at this point as the final measurement model provided excellent fit to the data (χ2 (df) = 235.77 (193), p = .02, CFI = .98, TLI =.98, RMSEA =.03, SRMR =.05).

**Structural Model**

Next, prospective paths and within-time covariances replaced factor covariances in the alcohol measurement model and resulted in a significant decrement in model fit (*p* = .01). Covariates were added next which resulted in a decrement in fit (*p* < .001). However, these paths were retained based on developmental theories of substance use which suggest accounting for our selected covariates. Lastly, modification indices suggested regressing W1 peer deviancy on W1 child externalizing symptoms. Given that this pathway was consistent with theories that posit that adolescents high in externalizing symptoms are more likely to affiliate with deviant peers (Rudolph et al., 2014), it was retained and resulted in significant improvement in model fit (*p* < .001). The final structural model fit the data well (χ2 (df) = 453.94 (334), *p* < .001, CFI = .96, TLI =.95, RMSEA =.03, SRMR =.06).

**Table 1**

*Interfactor Correlations of Correlated Measurement Model*

| *Variable* | *1* | *2* | *3* | *4* | *5* | *6* |
| --- | --- | --- | --- | --- | --- | --- |
| 1. W1 Maternal depression | - |  |  |  |  |  |
| 2. W1 Mother-child relationship | -.42 | - |  |  |  |  |
| 3. W1 Peer deviancy | .07 | -.19 | - |  |  |  |
| 4. W2-3 Peer deviancy | .17 | -.26 | .56 | - |  |  |
| 5. W2-3 Substance use | .13 | -.26 | .32 | .54 | - |  |
| 6. W4-6 Substance use | .05 | -.19 | .23 | .65 | .71 | - |
| *Note.* Non-substance use variable correlations were the same across models. Correlations greater or equal to \|.10\| in magnitude are significant at *p* < .05. W = Wave. | | | | | | |

**Figure 1**

*Latent Factor Structure for Correlated Measurement Models*


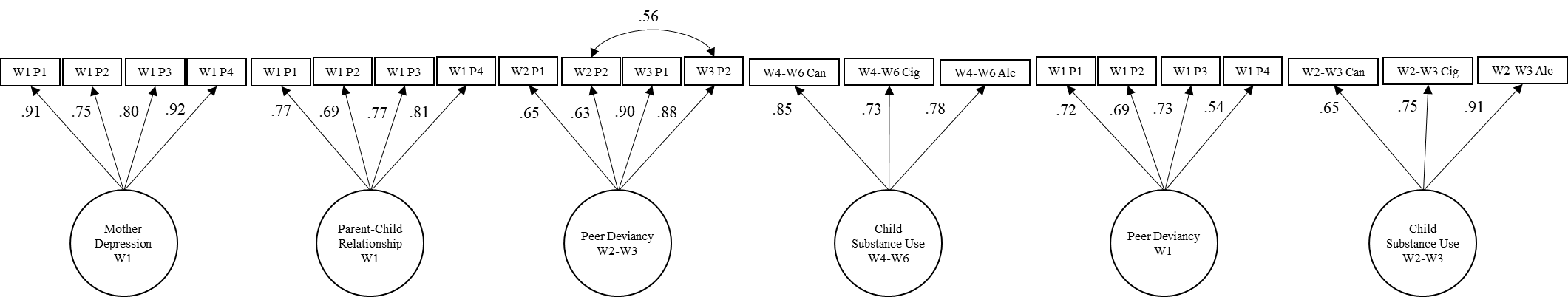


*Note.* Latent factor structure and standardized loading estimates are shown. Interfactor correlations were estimated between all latent factors but are shown in Table 1 to facilitate visual interpretatiom. Residual variances not shown for simplicity. W = Wave; P = Parcel; Alc = Alcohol; Can = Cannabis; Cig = Cigarette.
